# Supplementary material for: Recombinant Human TSH Fails to Induce the Proliferation and Migration of Papillary Thyroid Carcinoma Cell Lines
Source: Cancers (Basel). 2024 Jul 21;16(14):2604. doi: 10.3390/cancers16142604 (PMC11275150; doi:10.3390/cancers16142604)
Supplement: Supplementary file 1 [file cancers-16-02604-s001.zip › cancers-3086895-supplementary.pdf]

Gel and Blot Images

Supporting Data

Kalampounias G. et al, 2024

Original Images

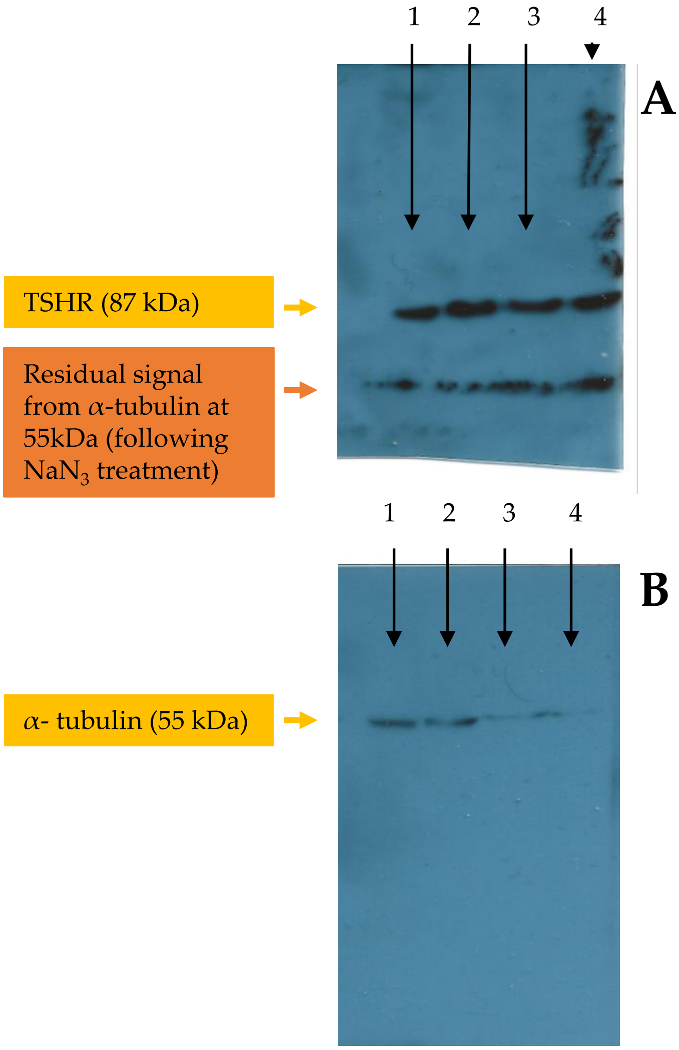

Figure 4d

K1	Naïve	Transformed	
Doxycycline	-	-	+
TSHR	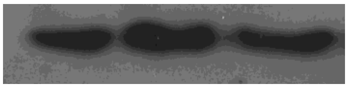		
Tubulin	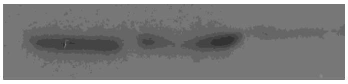		

A

B

1: K1 (non transformed/naïve)
2: K1-TSHR (transformed) DOX-untreated
3: K1-TSHR (transformed) DOX-treated
4: X

Image Processing

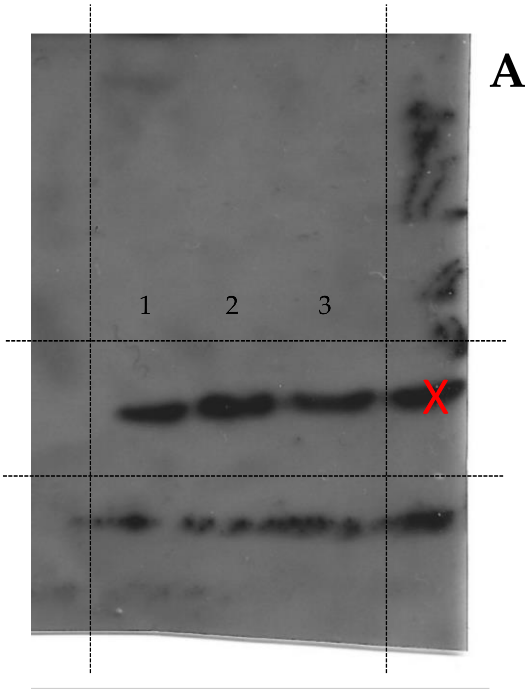

Image Processing

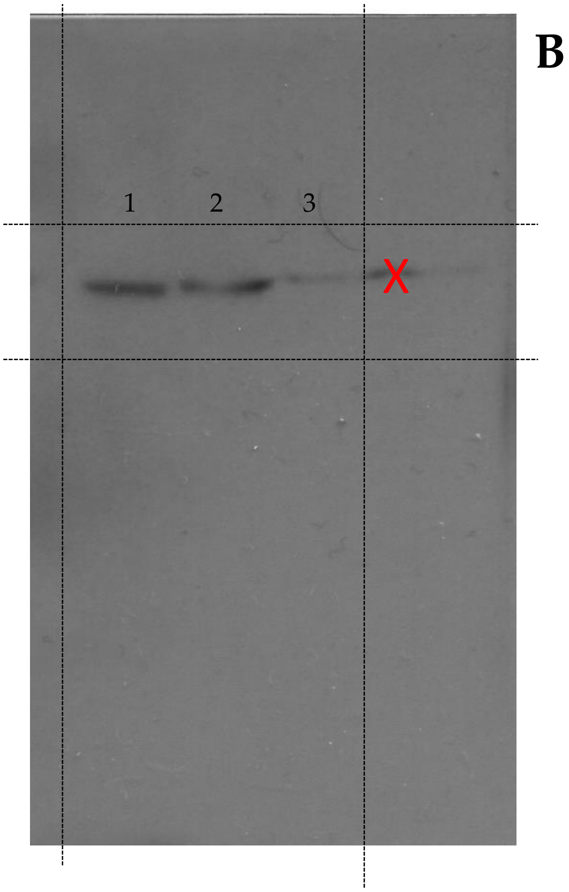

Original Images

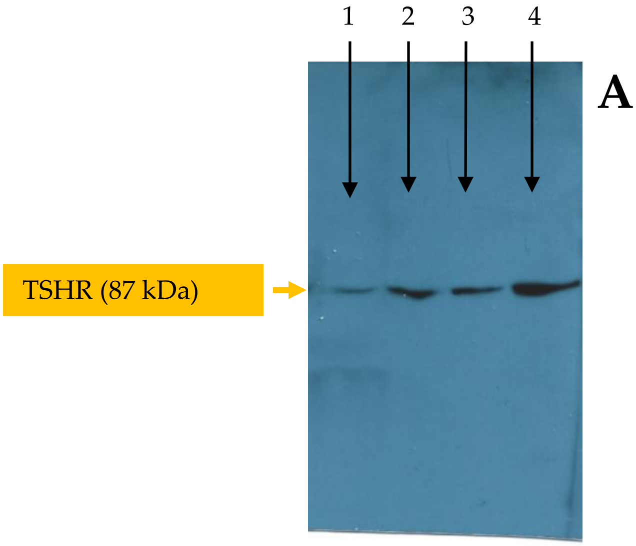

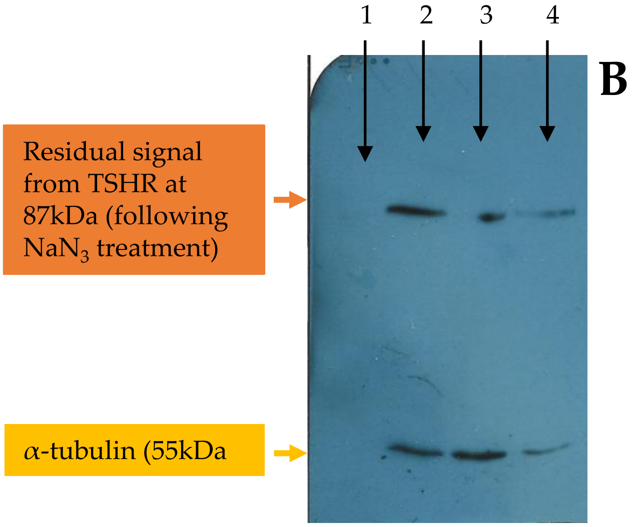

Figure 4e

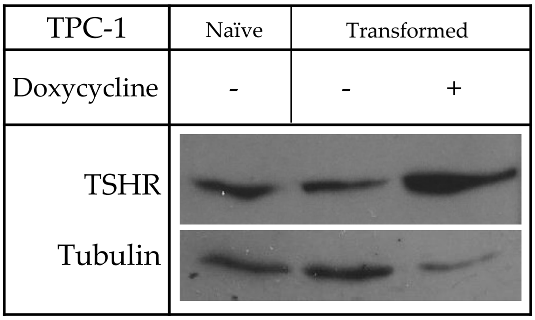

1: X
2: TPC-1 (non transformed/naïve)
3: TPC-1-TSHR (transformed) DOX-untreated
4: TPC-1-TSHR (transformed) DOX-treated

Image Processing

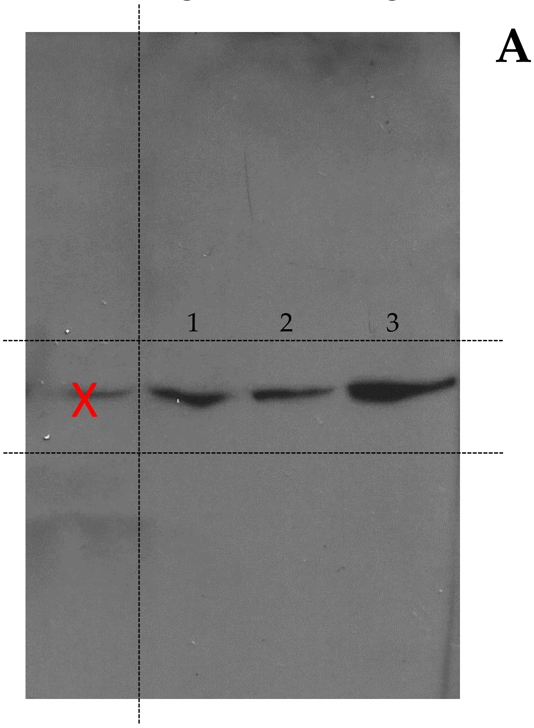

Image Processing

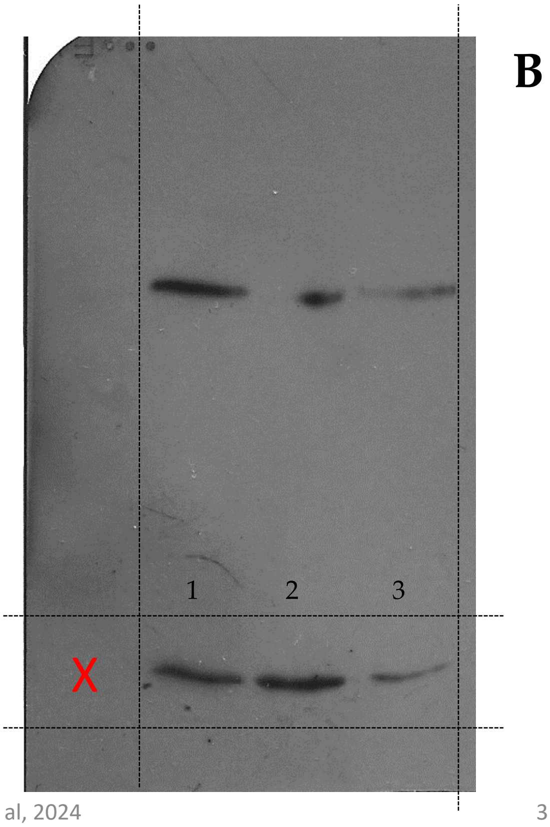

Original Image

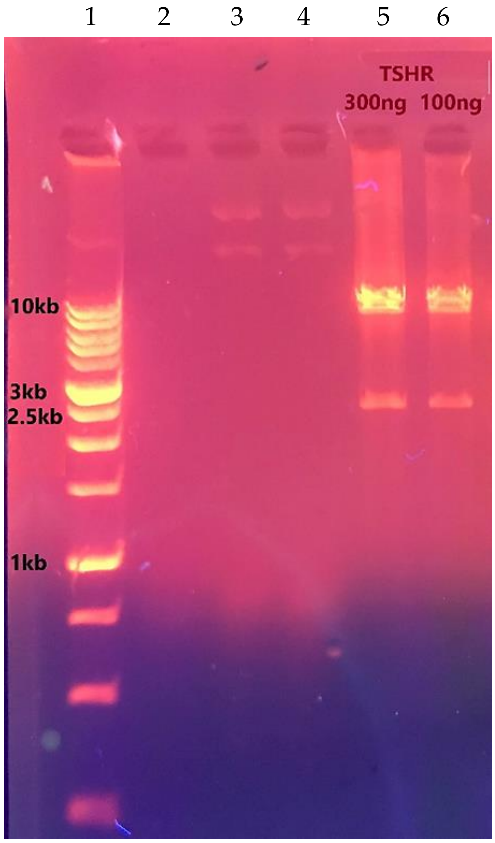

Image Processing

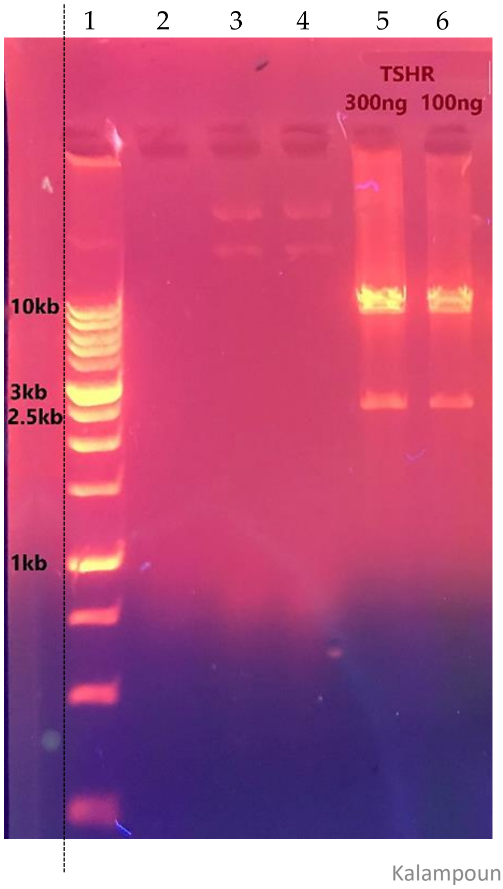

Figure A2a

	Ladder	Plasmids
m_{DNA}		**300 ng 100 ng**
10 kb<br>3 kb<br>2,5 kb<br>1kb	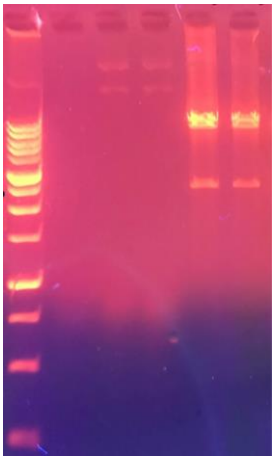	

1: DNA ladder
2: X
3: X
4: X
5: 300 ng of isolated TSHR plasmid
6: 100 ng of isolated TSHR plasmid

Figure A2b

	K1		TPC-1	
Transformed	-	+	-	+
TSHR	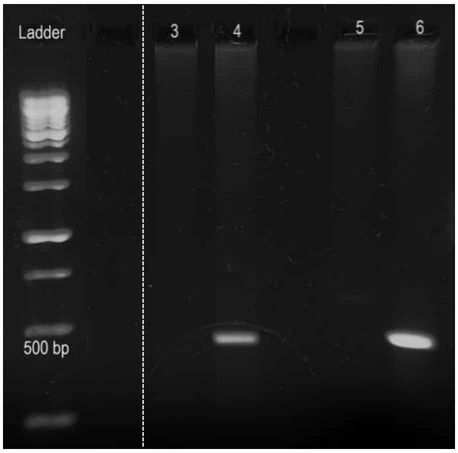			

Original Image

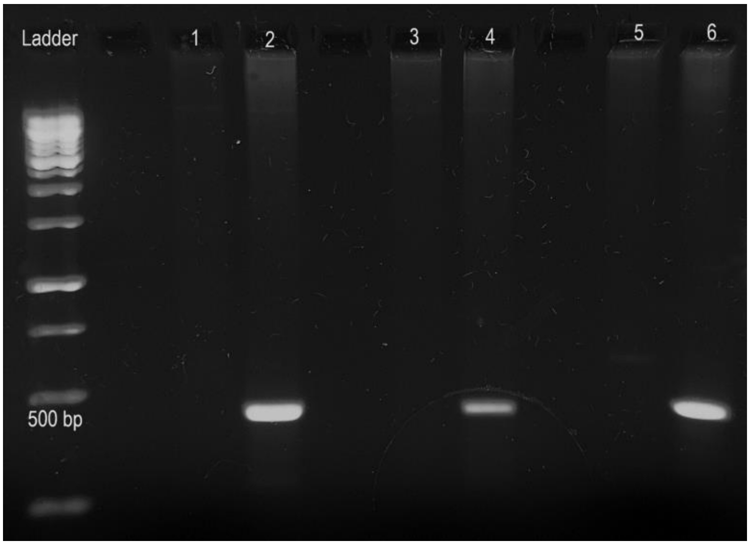

3: X
4: X
5: 300 ng of isolated TSHR plasmid
6: 100 ng of isolated TSHR plasmid

Image Processing

I II III IV V VI VII VIII IX X

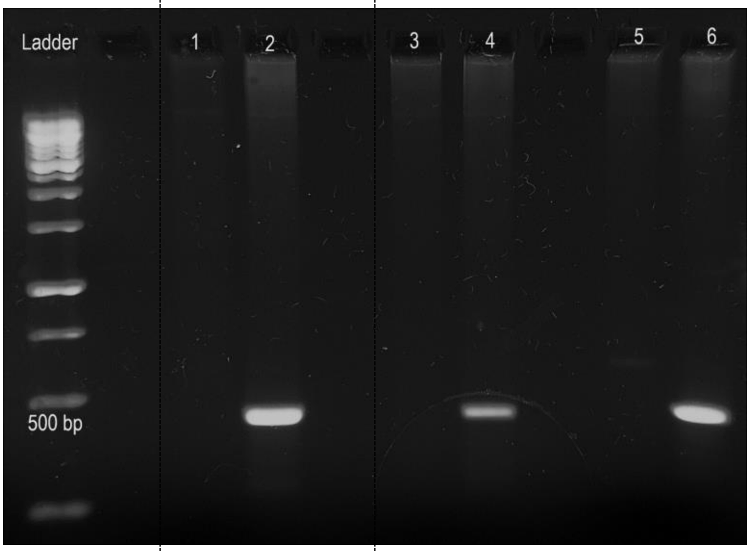

I: DNA ladder
II: not loaded
III: X
IV: X
V: not loaded
VI: (3) untransformed K1
VII: (4) transformed K1 (K!-TSHR)
VIII: not loaded
IX: (5) untransformed TPC-1
X: (6) transformed TPC-1 (TPC-1-TSHR)

Notes

BLOT IMAGES

- The films were scanned, and the images were transformed int grayscale pictures using ImageJ.
- The only adjustments made were Brightness and Contrast alterations, which were used to create the images appearing in the figures. For quantification of the signal, no adjustment had been made, as not to change the bit depth and intensity.
- The normalization was performed using the 'gels' tool by ImageJ.
- Some images are cropped, and every time cropping was performed, we present it here using arrows and lines.
- During SDS-PAGE molecular weight markers were used; based on their position on the membrane we annotate here the observed molecular weight of each polypeptide detected.
- Residual signal is visible in some images, and it is a result of a previous incubation of the membrane with HRP-linked antibodies. We did not perform stripping with β-ME on the membrane, to avoid protein loss; instead, we used a solution containing a very low NaN_3 concentration to deactivate the HRP of the previous antibodies and thus, avoid oversaturation of the films. Even though the proteins on the membrane are relatively distant (87 kDa vs 55 kDa) due to tubulin's abundance we used the mild sodium azide solution to obtain better results through neutralization of its signal.
- Regarding tubulin detection, a few seconds of film exposure were necessary while to adequately detect the TSHR, several minutes were required. This time difference demanded tubulin signal deactivation each time the membrane had probed with anti-tubulin antibodies.
- Every cropping, alteration or adjustment performed, did not affect the image's content or meaning.

AGAROSE GEL IMAGES

- The plasmid electrophoresis gel was photographed during ethidium bromide excitation on a transilluminator.
- The PCR product electrophoresis was photographed using a camera-mount transilluminator following ethidium bromide excitation by UV light.
- Every cropping, alteration or adjustment performed, did not affect the image's content or meaning.
